# A six-microRNA signature in plasma was identified as a potential biomarker in diagnosis of esophageal squamous cell carcinoma

**DOI:** 10.18632/oncotarget.16519

**Published:** 2017-03-23

**Authors:** Xin Zhou, Wei Wen, Jun Zhu, Zebo Huang, Lan Zhang, Huo Zhang, Lian-Wen Qi, Xia Shan, Tongshan Wang, Wenfang Cheng, Danxia Zhu, Yin Yin, Yan Chen, Wei Zhu, Yongqian Shu, Ping Liu

**Affiliations:** ^1^ Department of Oncology, First Affiliated Hospital of Nanjing Medical University, Nanjing 210029, PR China; ^2^ Department of Thoracic Surgery, First Affiliated Hospital of Nanjing Medical University, Nanjing 210029, PR China; ^3^ Department of Radiation Oncology, Jiangsu Cancer Hospital, Nanjing 210009, PR China; ^4^ State Key Laboratory of Natural Medicines and Department of Pharmacognosy, China Pharmaceutical University, Nanjing 210009, PR China; ^5^ Department of Respiration, The Affiliated Jiangning Hospital of Nanjing Medical University, Nanjing 210000, PR China; ^6^ Department of Gastroenterology, First Affiliated Hospital of Nanjing Medical University, Nanjing 210029, PR China; ^7^ Department of Oncology, The Third Affiliated Hospital of Soochow University, Changzhou 213003, PR China; ^8^ Department of Obstetrics and Gynecology, First Affiliated Hospital of Nanjing Medical University, Nanjing 210029, PR China; ^9^ Department of Emergency, First Affiliated Hospital of Nanjing Medical University, Nanjing 210029, PR China; ^10^ Cancer Center of Nanjing Medical University, Nanjing 210029, PR China

**Keywords:** miRNA, esophageal squamous cell carcinoma, diagnosis, exosomes, TCGA

## Abstract

The differential expression of microRNAs (miRNAs) in plasma of esophageal squamous cell carcinoma (ESCC) patients may serve as a diagnostic biomarker. A four-stage study was conducted to identify plasma miRNAs with potential in detecting ESCC. Exiqon panels (2 ESCC pools vs. 1 normal control (NC) pool) were applied in the screening phase to obtain miRNA profiles. The identified miRNAs were further evaluated through training (36 ESCC VS. 42 NCs) and testing stages (101 ESCC VS. 113 NCs) with qRT-PCR assays. A six-miRNA signature including up-regulated miR-106a, miR-18a, miR-20b, miR-486-5p, miR-584 and down-regulated miR-223-3p in ESCC was identified. The signature could accurately discriminate ESCC patients from NCs with areas under the receiver operating characteristic curve of 0.935, 0.959 and 0.966 for the training, testing and the additional validation stage (41 ESCC VS. 50 NCs), respectively. MiR-106a and miR-584 were significantly up-regulated in tumor tissues with qRT-PCR assays. And miR-584 was also up-regulated in ESCC tissues from TCGA database. In addition, exosomal miR-223-3p and miR-584 were consistently dysregulated with those in plasma and could also act as biomarkers in diagnosis of ESCC. In conclusion, we identified a six-miRNA signature in plasma which could act as a non-invasive biomarker in detection of ESCC.

## INTRODUCTION

Esophageal cancer is one of the most common malignancies with high mortality worldwide [1]. According to the pathological characteristics, esophageal cancer is mainly classified as esophageal squamous cell carcinoma (ESCC) and esophageal adenocarcinoma. Occupying more than 90% of esophageal cancer, ESCC is one of the most aggressive carcinomas of the gastrointestinal tract in Asian countries [2]. It was estimated that about 477,900 new diagnoses and 375,000 deaths occurred in China in 2015 [3]. Despite increased understanding of the molecular and clinical characteristics of ESCC as well as recent improvements in surgical techniques and perioperative management, the prognosis of ESCC is still poor, leading to an overall 5-year survival rate of 25–30% [4–9]. However, the 5-year survival rate of ESCC patients with an early stage could increase to 85% [10]. Therefore, many efforts have been made on early detection and intervention of the disease to increase the possibility of curable treatment and thus prolong the survival of ESCC patients. Nowadays, endoscopic or surgical biopsy is the diagnostic standard of ESCC, but the invasiveness, high cost and potentially subjective discrepancy caused by different operators might limit its effectiveness in screening ESCC in large-scale populations. Non-invasive tumor markers such as squamous cell carcinoma antigen (SCC) and carcinoembryonic antigen (CEA) did not show sufficient sensitivity and specificity for early diagnosis of ESCC [11, 12]. Hence, novel and reliable biomarkers to detect ESCC are urgently needed for early intervention with the potential to reduce mortality of the disease.

MicroRNAs (miRNAs) are small and non-coding RNAs (18–25 nucleotides in length) which could negatively regulate gene expression by translational repression or degradation of the target mRNAs [13, 14]. Numerous studies have indicated that dysregulation of miRNAs is involved in the tumorigenesis and progression of various cancers [15, 16]. Recently, many studies demonstrated that miRNAs were detectable in plasma/serum and could act as non-invasive biomarkers for diagnosis or prognosis of cancer [17, 18]. These findings have opened up a new and promising filed in the screening and monitoring of cancer patients. Since the first study on the levels of serum miRNAs in ESCC patients was reported in 2010 by Zhang et al. [19], several studies have investigated the differential expression of circulating miRNAs and explored the potential application of the biomarkers in ESCC [20]. However, the results were not consistent between the studies partly due to different research methods and tested populations. And most studies only focused on some specific miRNAs, while few studies comprehensively explored circulating miRNA profiles in ESCC.

In the present study, we conducted plasma miRNA profiles with Exiqon miRNA qPCR panel followed by multiple-stage validation to identify potential plasma miRNAs for diagnosis of ESCC. In addition, the identified miRNAs were also verified in tissues of ESCC. Moreover, exosomal miRNAs were further explored to evaluate the potential form of the miRNAs in plasma which might be useful in the detection of ESCC.

## RESULTS

### Clinical characteristics of subjects

**Figure 1:**
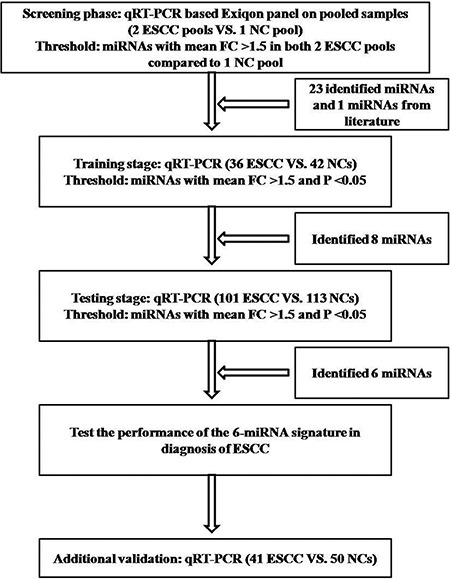
Overview of the experiment design ESCC: esophageal squamous cell carcinoma; NC: normal control; FC: fold change.

The study was divided into four stages to determine whether the differently expressed miRNAs in plasma of ESCC patients could aid the detection of ESCC (Figure 1). A total of 383 subjects, including 178 ESCC patients and 205 normal controls (NCs), were enrolled in our study. After the initially screening stage, the ESCC patients and NCs were divided into three stages: the training stage, the testing stage and the additional validation stage. As shown in Table 1, no significant difference was observed in age or gender distribution between cases and controls in any stage (*p*-values > 0.05).

**Table 1:** Characteristics of 178 ESCC patients and 205 normal controls enrolled in the study

Variables	Screening phase (*n* = 30)		Training cohort (*n* = 84)		Testing cohort (*n* = 214)		Additional validation cohort (*n* = 99)	
	Cases (%)	Controls (%)	Cases (%)	Controls (%)	Cases (%)	Controls (%)	Cases (%)	Controls (%)
**Number**	20	10	36	42	101	113	41	50
**Gender**								
Male	14 (70)	6 (60)	25 (69)	27 (64.3)	77 (76.2)	75 (66.4)	24 (58.5)	26 (52)
Female	6 (30)	4 (40)	11 (31)	15 (35.7)	24 (23.8)	38 (33.6)	17 (41.5)	24 (48)
**Age**								
< 65	13 (65)	5 (50)	20 (55.6)	31 (73.8)	65 (64.4)	81 (71.2)	25 (61)	34 (68)
≥ 65	7 (35)	5 (50)	16 (44.4)	11 (26.2)	36 (35.6)	32 (28.8)	16 (39)	16 (32)
**Smoking**								
Smoker	9 (45)		14 (38.9)		39 (38.6)		17 (41.5)	
Non-smoker	8 (40)		15 (41.7)		47 (46.5)		22 (53.7)	
NA	3 (15)		7 (19.4)		15 (14.9)		2 (4.8)	
**Drinking**								
Drinker	3 (15)		7 (19.4)		23 (22.7)		7 (17.1)	
Non-drinker	11 (55)		23 (63.9)		71 (70.3)		26 (63.4)	
NA	6 (30)		6 (16.7)		7 (7)		8 (19.5)	
**Differential**								
well	10 (50)		19 (52.8)		48 (47.5)		19 (46.3)	
poor	7 (35)		14 (38.9)		42 (41.6)		14 (34.2)	
NA	3 (15)		3 (8.3)		11 (10.9)		8 (19.5)	
**Location**								
Up	2 (10)		3 (8.3)		6 (5.9)		4 (9.8)	
Middle	9 (45)		15 (41.7)		31 (30.7)		16 (39)	
Down	7 (35)		14 (38.9)		49 (48.5)		15 (36.6)	
NA	2 (10)		4 (11.1)		15 (14.9)		6 (14.6)	
**TNM stage**								
I	8 (40)		15 (41.7)		46 (45.5)		18 (43.9)	
II	7 (35)		14 (38.9)		32 (31.7)		18 (43.9)	
III	5 (25)		7 (19.4)		23 (22.8)		5 (12.2)	

### Discovery of candidate miRNAs from the screening phase

To identify candidate miRNAs in plasma of ESCC patients in the screening phase, we conducted miRNAs profiles from 2 ESCC and 1 NC pooled samples using Exiqon miRCURY-Ready-to-Use-PCR-Human-panel-I + II-V1.M which could detect 168 miRNAs with relatively high abundance in plasma/serum. A miRNA was omitted if its cycle threshold (Ct) value was larger than 37 or 5 higher than the negative control in the panel. Nevertheless, a miRNA was considered as a candidate miRNA if it showed more than 1.5-fold or less than 0.67-fold altered expression in both 2 pooled ESCC samples compared to the NC pool sample. Thus, 17 up-regulated miRNAs and 6 down-regulated miRNAs were subjected to the further analysis (Supplementary Table 1 online). In addition, another miRNA (miR-21) from the previous literature was also chosen to the further validation stage [21].

### Confirmation of miRNAs in plasma by qRT-PCR

**Figure 2:**
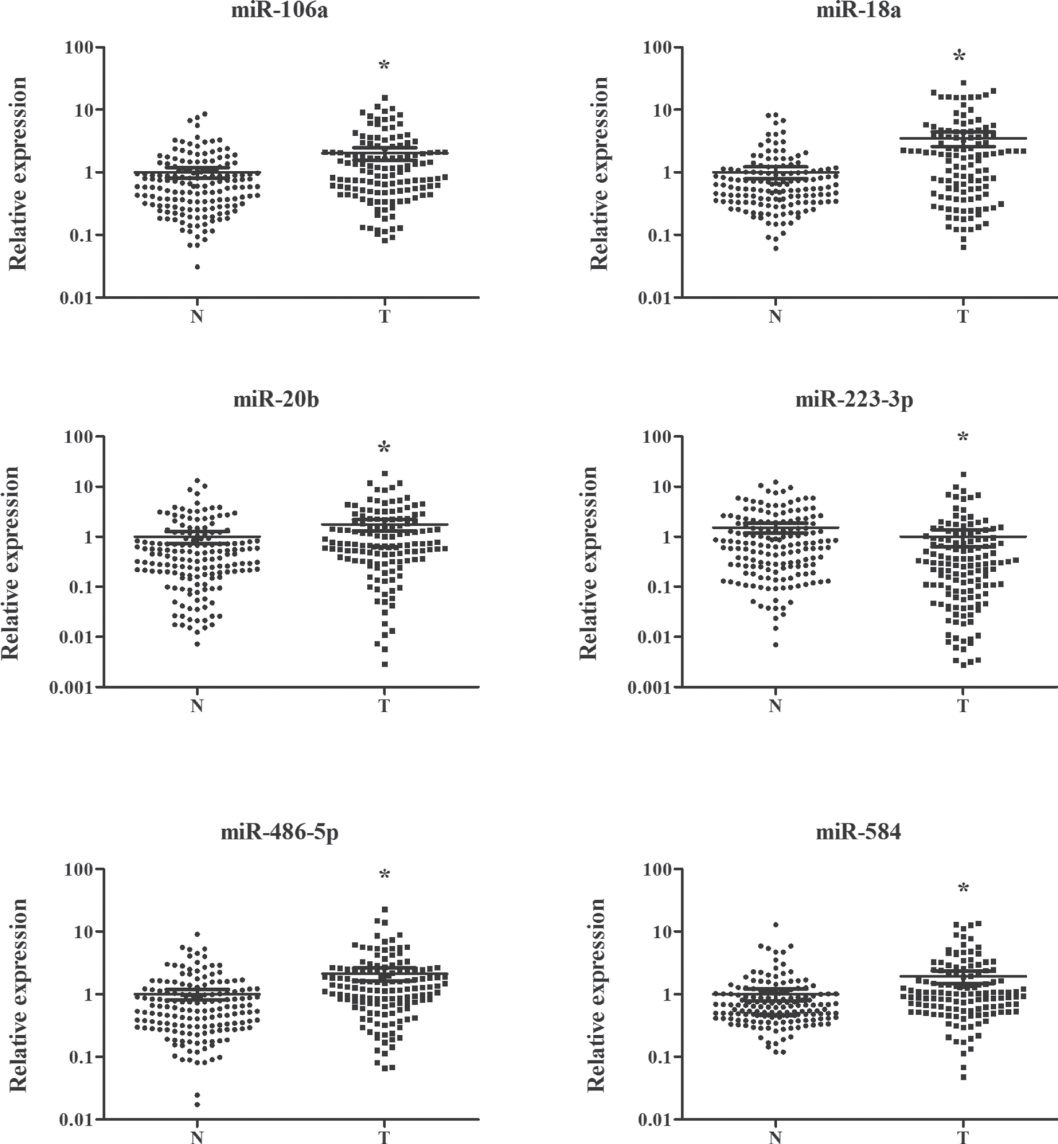
Expression levels of six miRNAs in plasma of 137 ESCC patients and 155 controls (in the training and testing stages) N: normal controls; T: tumor. Horizontal line: mean with 95% CI. *P-value < 0.05.

The expression of 24 miRNAs obtained from the screening phase was analyzed in plasma samples from 36 ESCC patients and 42 NCs in the training stage. To explore the potential effect of hemolysis on plasma samples, two hemolysis markers (miR-16 and miR-451) were also evaluated [22]. However, the expression levels of miR-1228, miR-16 and miR-451 were not altered between plasma samples of ESCC and NCs (Supplementary Figure 1 online). Relative to the endogenous control miR-1228, 8 dysregulated miRNAs in the training stage were subjected to the testing stage. In the larger cohort, 6 of 8 miRNAs were consistently dysregulated with those in the training stage (Table 2; the other miRNAs were shown in the Supplementary Table 2 online). Combined results of the two stages showed that miR-106a, miR-18a, miR-20b, miR-486-5p and miR-584 were significantly up-regulated, while miR-223-3p was down-regulated in plasma of ESCC patients compared with NCs (Table 2; Figure 2).

**Table 2:** Expression levels of the six plasma miRNAs in the training and testing stages (presented as mean ± SD; ΔCT, relative to miR-1228)

miRNA	Training stage				Testing stage				Combined	
	Cases	Controls	FC	*P* value	Cases	Controls	FC	*P* value	FC	*P* value
miR-106a	6 ± 1.78	6.75 ± 1.61	1.67	0.048	5.8 ± 1.64	6.62 ± 1.38	1.77	< 0.001	1.72	< 0.001
miR-18a	9.93 ± 1.98	10.81 ± 1.54	1.83	0.038	9.62 ± 2.12	10.53 ± 3.87	1.87	< 0.001	1.84	< 0.001
miR-20b	7.81 ± 2.58	8.87 ± 2.24	1.93	0.032	7.73 ± 2.23	8.54 ± 2.3	1.75	0.003	1.77	< 0.001
miR-223-3p	3.32 ± 2.89	1.44 ± 2.42	0.27	0.003	2.59 ± 2.66	1.36 ± 2	0.43	0.001	0.38	< 0.001
miR-486-5p	0.78 ± 1.51	1.41 ± 1.73	1.55	0.048	0.26 ± 1.59	1.42 ± 1.43	2.24	< 0.001	2.01	< 0.001
miR-584	9.08 ± 1.13	9.92 ± 1.33	1.8	< 0.001	9.26 ± 1.63	9.86 ± 1.01	1.52	0.001	1.56	< 0.001

### Diagnostic value of plasma miRNAs for ESCC

The diagnostic performance of the six plasma miRNAs was evaluated by receiver operating characteristic (ROC) curves. The data from the training and testing stage were combined to calculate the optimal cutoff values for miR-106a, miR-18a, miR-20b, miR-223-3p, miR-486-5p and miR-584. The corresponding areas under the ROC curve (AUCs) were 0.639 (95% confidence interval (CI): 0.574–0.703), 0.661 (95% CI: 0.586–0.735), 0.627 (95% CI: 0.562–0.691), 0.649 (95% CI: 0.586–0.711), 0.688 (95% CI: 0.627–0.749) and 0.659 (95% CI: 0.595–0.723) for miR-106a, miR-18a, miR-20b, miR-223-3p, miR-486-5p and miR-584, respectively (Supplementary Figure 2 online).

**Figure 3:**
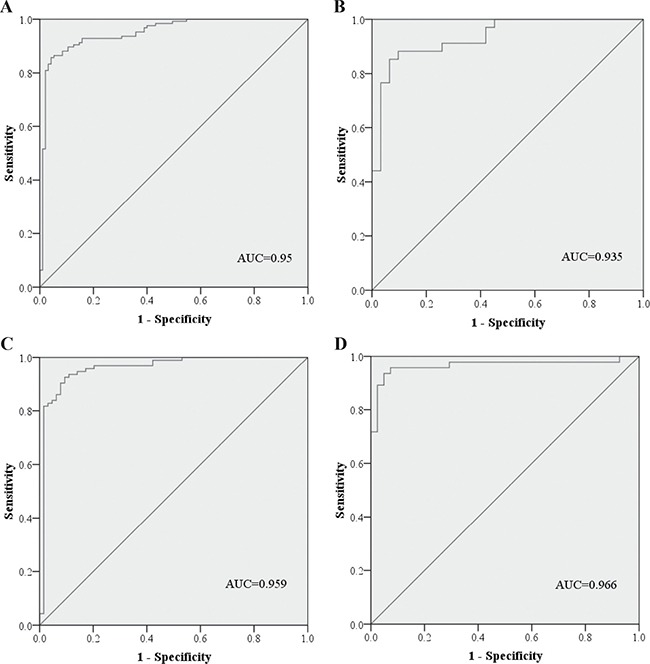
Receiver-operating characteristic (ROC) curve analyses of the six-miRNA signature to discriminate ESCC patients from normal controls (**A**) the combined two cohorts of training and testing stages (137 ESCC VS. 155 NCs); (**B**) training stage (36 ESCC VS. 42 NCs); (**C**) testing stage (101 ESCC VS. 113 NCs); (**D**) additional validation stage (32 ESCC VS. 18 NCs). ESCC: esophageal squamous cell carcinoma; NC: normal control. AUC: areas under the curve.

The predicted probability of detection with ESCC from the logistic regression model was calculated with the equation: Logit(P) = 17.54 - 0.024 × miR-106a - 0.403 × miR-18a - 0.051×miR-20b + 1.715 × miR-223-3p - 1.502 × miR-486-5p - 1.844×miR-584. The diagnostic utility of the six-miRNA signature was also confirmed in the combined two stages by ROC curves and yielded the AUC of 0.95 (95% CI: 0.921–0.979; sensitivity = 85.7% and specifity = 95.8%; Figure 3A). Similarly, the six-miRNA signature could also discriminate ESCC patients from NCs in the training (AUC = 0.935, 95% CI: 0.878–0.993; sensitivity = 85.3% and specifity = 93.5%) and testing stage (AUC = 0.959, 95% CI: 0.924–0.994; sensitivity = 92.5% and specifity = 90.6%), respectively (Figure 3B and 3C).

The diagnostic value of the six-miRNA signature was further verified in another independent validation stage including 41 ESCC patients and 50 NCs. MiR-106a, miR-18a, miR-20b, miR-486-5p and miR-584 were significantly up-regulated and miR-223-3p was down-regulated in plasma of ESCC patients which was identical to the results in the training and testing stage (Supplementary Table 3 online). By using the same equation, the AUC of the miRNA signature in detecting ESCC was 0.966 (95% CI: 0.923–0.995) with sensitivity of 93.5% and specifity of 95.1%.

The association of the six plasma miRNAs with clinical parameters (gender, age, smoking status, drinking status, tumor differentiation, location and stage) was analyzed using data of all 178 ESCC patients. But no significant correlation was found between the plasma miRNAs or miRNA signature and ESCC patients’ characteristics (*p*-values > 0.05). Moreover, the performance of the six-miRNA signature in discriminating 178 ESCC patients with different TNM stages from NCs was performed by ROC analyses. The corresponding AUCs for ESCC patients with stage I, II and III were 0.92 (95% CI: 0.834–0.992), 0.925 (95% CI: 0.87–0.979) and 0.936 (95% CI: 0.9–0.972), respectively (Supplementary Figure 3 online).

### Evaluation of the miRNAs in tissue samples

**Figure 4:**
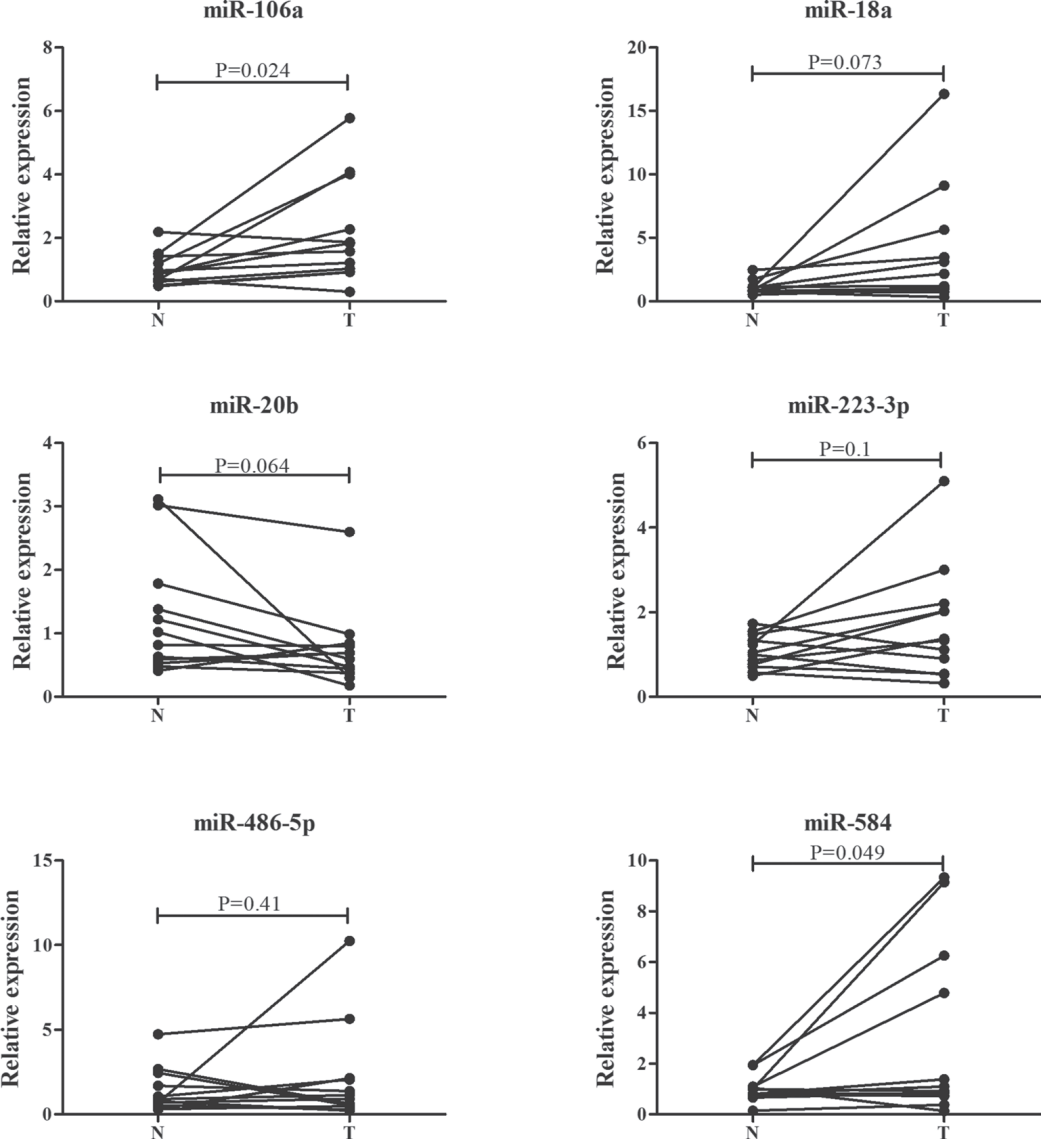
Expression of the six selected miRNAs in the 12 pairs of ESCC and matched normal oesophageal tissues T: tumor. N: control.

To explore the expression of the six miRNAs in tissue ssamples, 12 pairs of ESCC and matched normal oesophageal tissues were analyzed. As shown in Figure 4, miR-106a and miR-584 but not the other four miRNAs were significantly up-regulated in tumor tissues. In addition, the miRNA expression microarray data of 9 ESCC tissues and matched normal tissues from the TCGA database were assessed. And miR-584 was found to be consistently up-regulated in ESCC tissues (Supplementary Figure 4 online).

### Identification of miRNAs in exosomes

**Figure 5:**
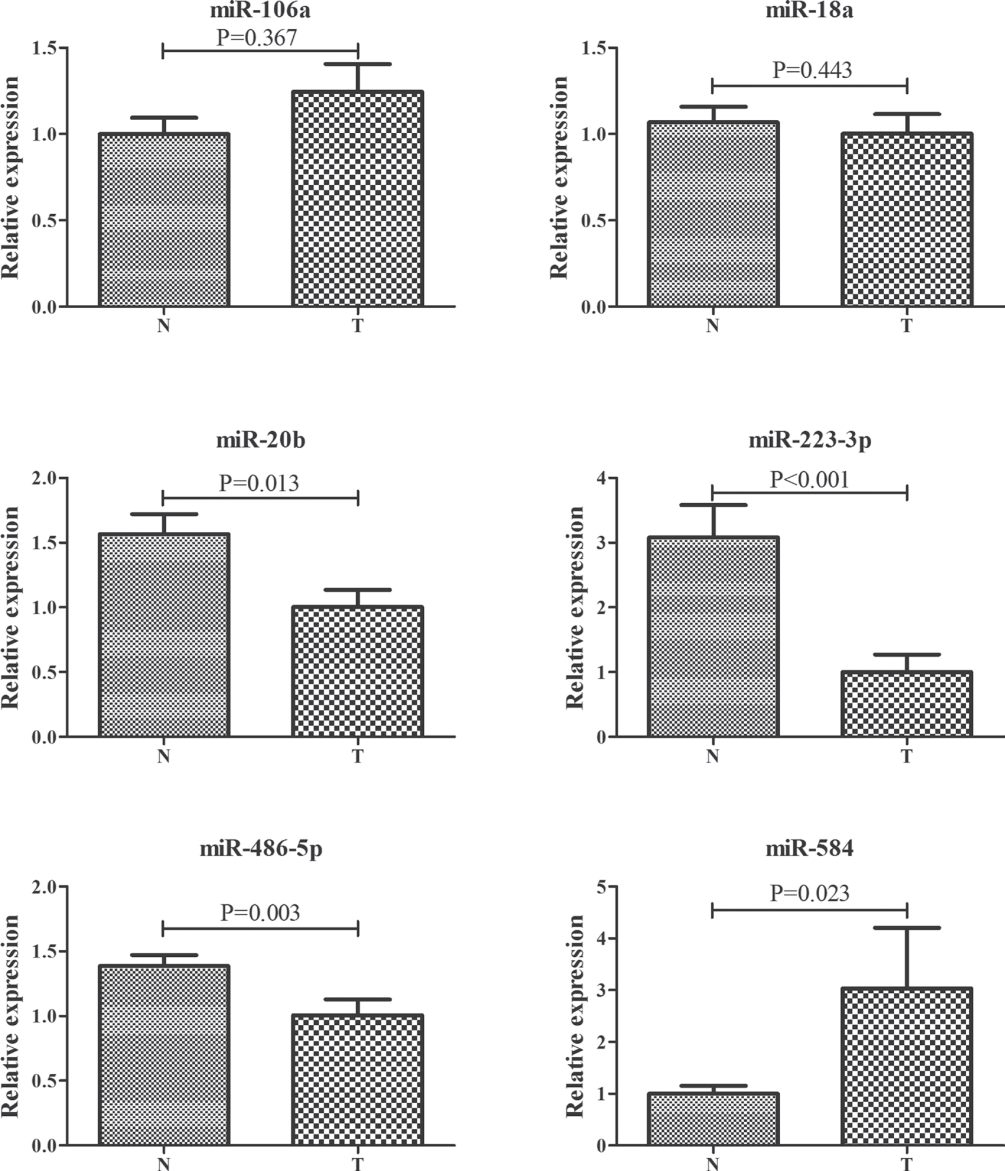
Expression of the six miRNAs in plasma exosomes from 30 ESCC patients and 34 NCs T: tumor. N: control. Error bar: standard error.

In addition, the expression of the six miRNAs was examined in plasma exosomes from 30 ESCC patients and 34 NCs to evaluate the potential form of the miRNAs in plasma of ESCC patients. As shown in Figure 5, significant dysregulation of exosomal miR-223-3p and miR-584 was consistent with those in plasma. And miR-584 was the only miRNA showed the consistent tendency in plasma, tissue and exosomes. While miR-20b and miR-486-5p were significantly down-regulated in exosomes from ESCC patients compared with NCs.

Moreover, the diagnostic value of exosomal miR-223-3p, miR-584 and the signature of the two exosomal miRNAs was evaluated and compared with those in plasma from the same subjects (Supplementary Figure 5 online). The AUC for exosomal miR-223-3p was 0.854 (95% CI: 0.754–0.954) which was higher than plasma miR-223-3p (0.717, 95% CI: 0.59–0.843). But AUCs for exosomal miR-584 (0.665, 95% CI: 0.527–0. 803) and plasma miR-584 (0.648, 95% CI: 0.513–0. 783) were approximate. The capacity of the signature of the two exosomal miRNAs (AUC = 0.881, 95% CI: 0.798–0.964) and the two plasma miRNAs (AUC = 0.874, 95% CI: 0.788–0.96) in detecting ESCC was similar.

## DISCUSSION

During the last decades, many studies have demonstrated that miRNAs could contribute to tumorgenesis and development of various cancers and might be a treasure for cancer diagnosis and therapy [23]. In 2008, Mitchell et al. firstly reported that miR-141 could be stably detected in serum and could act as a potential diagnostic marker for prostate cancer (PC) [17]. Subsequently, the potential use of circulating miRNAs as novel biomarkers was widely explored in various cancers, such as lung cancer, colorectal cancer, gastric cancer and breast cancer [24–27].

Concerning ESCC, several studies have indicated the potential value of circulating miRNAs in diagnosis of the disease [20]. But most studies only focused on some specific miRNAs, few studies explored circulating miRNA profiles using the comprehensive array-based approach in ESCC. Among these reports, four studies comprehensively analyzed miRNA profiles in serum of ESCC patients [19, 28–30]. However, only one study conducted miRNA profiles in plasma. In the study, only plasma miR-25 was identified as a potential diagnostic marker out of the eight candidate miRNAs from miRNA array-based approaches [31]. Recent studies showed that profiles of circulating miRNAs could be different in plasma and serum [32]. This might also contribute to the inconsistency of the results between laboratories. Thus, we conducted the comprehensive miRNA profiles followed by multiple-stage validation to identified candidate plasma miRNAs with potential application in detecting ESCC.

Here, we performed a four-stage study to identify plasma miRNAs which could aid in detecting ESCC. Exiqon miRNA qPCR panels could measure 168 abundantly expressed miRNAs in plasma/serum with better sensitivity and linearity than TaqMan platform [33]. Thus, the panel was applied to conduct miRNA profiles in the screening phase. MiR-21, a well-known onco-miRNA and reported to be up-regulated in ESCC in some previous studies [21, 34–36], was also selected as a candidate miRNA for further analysis. Hu et al. recently investigated global circulating miRNA profiles of control individuals and patients with eight types of cancers (including esophageal cancer) and found that miR-1228 might act as a promising stable endogenous control for quantifying circulating miRNAs in cancer patients [37]. Confirmation in the training stage, plasma miR-1228 did not show different expression between ESCC patients and NCs and was used as the endogenous control in our study. To assess the effect of potential hemolysis during sampling on the results, plasma miR-16 and miR-451 [22] were assessed with similar expression levels between plasma samples from cases and NCs. In the following training and testing stages, a miRNA signature including five up-regulated (miR-106a, miR-18a, miR-20b, miR-486-5p and miR-584) and one down-regulated (miR-223-3p) plasma miRNAs were identified and could act as a biomarker in diagnosis of ESCC. Though discovered in the screening phase, miR-25 was not assessed as a candidate miRNA in the training stage in our study. Similarly, miR-21 was identified through the training stage but not confirmed in the testing stage with larger cohort. The additional validation stage further demonstrated the reliability of the six-miRNA signature in diagnosis of ESCC. Analysis of the combined data of the three stages showed that the miRNA signature could also accurately discriminate ESCC patients with stage I, II or III from NCs. The six miRNAs were also assessed in ESCC tissues. However, only miR-106a and miR-584 were consistently up-regulated in ESCC tissues. In addition, by analyzing the genome-wide miRNA expression profiles from TCGA, we found that miR-584 was consistently up-regulated in ESCC tissues. Anyway, the results are warranted to be validated in more ESCC tissue samples.

Among the six miRNAs, miR-18a was one of the key oncogenic components of the miR-17-92 cluster. Hirajima et al. reported that expression of miR-18a was significantly higher in ESCC tissues, ESCC cell lines and plasma of ESCC patients than normal controls. In addition, plasma miR-18a was decreased in postoperative samples compared with preoperative samples. The study indicated that plasma miR-18a might act as a biomarker for cancer detection and monitoring tumor dynamics in ESCC patients [38]. High expression of miR-18a could promote proliferation of ESCC cells and predict worse outcomes of ESCC patients [39, 40]. In our study, we also identified high expression of miR-18a in plasma of ESCC patients. Although not show significantly different expression in ESCC tissues, the results from PCR assay (*P* = 0.073) and TCGA (*P* = 0.053) both indicated high expression of miR-18a in ESCC patients with borderline significance. Future studies with more ESCC tissue samples might better reveal the phenomena. As for miR-223-3p, controversial roles were found in ESCC. Up-regulated in serum and tissues of ESCC patients, miR-223-3p could inhibit tumour-suppressor gene F-box and WD repeat domain-containing 7 (FBXW7) and demonstrate a poor prognosis [19, 41, 42]. On the other hand, down-regulation of miR-223-3p was also found in ESCC tissues, and ectopic expression of the miRNA could decrease migration and invasion of ESCC cells [43, 44]. In the current study, we did not identify dysregulation of miR-223-3p in ESCC tissue with limited samples. But miR-223-3p was down-regulated in plasma of ESCC patients which was just opposite to the results of the miRNA in serum. The role and mechanisms of the miRNA in ESCC are needed to be further explored. Until now, no study evaluated the expression level of circulating miR-486-5p in ESCC. It was reported that miR-486-5p was down-regulated in ESCC tissues and could serve as a favorable predictor in prognosis of ESCC [43, 45]. In our study, we firstly assessed high expression of miR-486-5p in plasma but not tissue samples of ESCC. However, more studies are warranted to examine the role of miR-486-5p in ESCC. As members of the miR-106a-363 cluster, miR-106a and miR-20b were rarely studied in ESCC. Only one study analyzed the expression of miR-106a in tissues of 21 ESCC patients and found that it was decreased in patients who developed recurrent disease or had a tumor-related death. The findings were not solid due to the relatively small sample size. However, high expression of circulating miR-106a was found in other digestive tract cancers such as gastric cancer and colorectal cancer [46, 47] and could act as an onco-miRNA in various cancers [48–50]. These findings were consistent with our results that miR-106a was up-regulated in plasma and tissue of ESCC patients though not identified in TCGA database. As for miR-20b, it might play distinct roles in various cancers [51–54] and needed to be better explored in ESCC. The other miRNA, miR-584, consistently up-regulated in plasma and tissue of ESCC patients (also identified in TCGA), was firstly reported and could act as a potential marker in detecting ESCC in our study. However, future studies of these miRNAs in ESCC formation and development is warranted.

Circulating miRNAs were believed to be released or transported from cells during tumorigenesis and might reflect tumor itself or physiological response (e.g. inflammation) caused from the disease [55]. In our study, the PCR assays showed that miR-106a and miR-584 but not the other four miRNAs were consistently up-regulated in plasma and tissue of ESCC patients. The results might suggest that plasma miR-106a and miR-584 (especially miR-584; also identified in TCGA database) might be closely related with ESCC itself and have potential in monitoring dynamics of ESCC. The other four miRNAs might be associated with some specific response. Binding to proteins, such as the Argonaute 2 protein and high-density lipoprotein, or packaged into small membranous vesicles, such as exosomes, could protect miRNAs from degradation by endogenous ribonuclease activity in plasma/serum [56–58]. Thus, we evaluated the expression of exosomal miRNAs to identify the potential form of the six miRNAs in plasma of ESCC patients which might aid in detection of ESCC. Interestingly, miR-20b and miR-486-5p was down-regulated in exosomes of ESCC patients which was opposite to the expression levels of the two miRNAs in plasma. While exosomal miR-106a and miR-18a did not show different expression levels between ESCC patients and NCs. We assumed that the majority of the four miRNAs (especially miR-20b and miR-486-5p) might bind to proteins in plasma of ESCC patients. The other two miRNAs, miR-223-3p and miR-584, were consistently dysregulated in ESCC exosomes compared with those in ESCC plasma. Thus, the two miRNAs might be packaged by exosomes in plasma of ESCC patients. Considering the consistent expression level in tissue, plasma and exosomes of ESCC patients, we suggested that miR-584 might be packaged by exosomes and released into blood by ESCC tumors and could transfer information from tumor cells to recipient cells. In addition, the diagnostic capacity of the exosomal miR-223-3p and miR-584 was evaluated and compared with those in the same subjects in plasma of ESCC patients. ROC analyses showed that exosomal miR-223-3p had greater ability in discriminating ESCC patients and NCs than plasma miR-223-3p. The diagnostic performance of miR-584 and the signature of miR-223-3p and miR-584 in exosomes and plasma was similar. These also illustrated the potential use of exosomal miRNA as biomarkers in detecting ESCC. To better use the biomarkers in the future clinical, many efforts should be made.

Several limitations of the study should be considered. First, only 12 and 9 pairs of ESCC tissues were analyzed by PCR assays and from TCGA database separately in our study. Future studies with larger sample size might validate our results. Second, ESCC patients enrolled in our study all underwent oesophagectomy. The miRNA-signature identified in our study could act as a diagnostic marker for ESCC patients with stage I, II or III. But we did not evaluate the diagnostic performance in patients with metastasis. Third, the mechanism of exosomal miRNAs in ESCC needs to be further studied.

In conclusion, we identified a six-miRNA signature in the plasma of ESCC patients which could act as a non-invasive biomarker in detecting ESCC. However, more efforts should be made before the application of circulating miRNAs for the detection of ESCC in the future clinical.

## MATERIALS AND METHODS

### Study design, patients and samples

The study was approved by Institutional Review Boards of the First Affiliated Hospital of Nanjing Medical University, and each participant provided the written informed consent. Between 2011 and 2013, 178 histopathologically confirmed ESCC patients who underwent radical oesophagectomy at First Affiliated Hospital of Nanjing Medical University were enrolled in the study. Clinical characteristics for each patient were also recorded. Blood samples from 205 healthy donors who conducted routine health checkup at First Affiliated Hospital of Nanjing Medical University were used as NCs.

Blood sample from ESCC patients before initial treatment and healthy donors were collected with ethylenediaminetetraacetic acid (EDTA)-containing tubes (Becton, Dickinson and Company). Cell-free plasma was separated from blood samples within 6 hours after collection using a two-step protocol (350 RCF (reactive centrifugal force) for 10 min, 20,000 RCF for 10 min (Beckman Coulter, USA)) to prevent contamination by cellular nucleic acids. Plasma samples were then stored at −80°C for further processing. Twelve pairs of ESCC and matched normal oesophageal tissues were obtained and kept in liquid nitrogen.

Overall, we conducted a multiphase, case-control study to identify plasma miRNAs as surrogate markers for ESCC (Figure 1). In the initially screening phase, 2 ESCC pool samples and 1 NC pool sample (20 ESCC and 10 NCs plasma samples were randomly selected and per 10 samples were pooled as 1 pool sample) were subjected to Exiqon miRCURY-Ready-to-Use PCR-Human-panel-I+II-V1.M (Exiqon miRNA qPCR panel, Vedbaek, Denmark; 168 miRNAs) to identify differently expressed plasma miRNAs between the ESCC cases and NCs. The process of arrays and analyses was performed according to the previous study [24]. Latter, a validation analysis including the training and the testing stage with qRT-PCR assay was applied to refine the number of plasma miRNAs in the ESCC signature.

In the training stage, the miRNAs identified from the screening phase and one miRNA (miR-21) from the literature [21] were determined in samples of 36 ESCC and 42 NCs. Subsequently, the miRNAs validated through the training stage were further confirmed in plasma samples of 101 ESCC patients and 113 NCs in the testing phase. The ESCC miRNA-signature was further assessed in an additional validation cohort including 41 cases and 50 NCs.

In addition, the expression level of the miRNAs was also explored in the 12 pairs of ESCC and matched normal oesophageal tissues with qRT-PCR assays and 9 pairs of ESCC and matched normal tissues from the TCGA database (http://cancergenome.nih.gov/). Exosomal miRNAs were further assessed in 30 ESCC patients and 34 NCs.

### Isolation of exosomes

Exosomes were extracted from plasma samples using ExoQuick Exosome Precipitation Solution (System Biosciences, Mountain View, Calif) in accordance with the manufacturer's protocol. Briefly, precipitated from 400 μl plasma and100 μl ExoQuick exosome precipitation solutions, exosomes pellets were then dissolved in 200 μl of RNase-free water for further RNA extraction.

### RNA extraction

Total RNA was extracted from 200 μl plasma or exosomes using the mirVana PARIS Kit (Ambion, Austin, TX, USA) according to the manufacturer's protocol. Trizol (Invitrogen, Carlsbad, CA, USA) was used to extract total RNA from tissue samples in accordance with instructions. Total RNA was eluted into 100 μl of RNase-free water. The purity and concentration of all RNA samples were quantified using Nanodrop 2000 thermo scientific spectrophotometer (NanoDrop Technologies, Wilmington, DE, USA).

### Quantitative reverse transcription polymerase chain reaction (qRT-PCR)

The expression level of each miRNA was quantified in triplicate using SYBR Green miScript PCR system (TaKaRa, Dalian, China) on 7900HT real-time PCR system (Applied Biosystems, Foster City, CA, USA). Bulge-Loop^™^ miRNA qRT-PCR Primer Set (RiboBio, Guangzhou, China) was applied to amplify miRNAs. The process of RT and PCR were performed as previously described [24, 59].

Briefly, RT reactions were conducted at 42°C for 60 min followed by 70°C for 10 min. The qRT-PCR reactions were carried out at 95°C for 20 sec, followed by 40 cycles of 95°C for 10 sec, 60°C for 20 sec and then 70°C for 10 sec. The melting curve analysis was added to assess the specificity of PCR products.

The expression levels of miRNAs from plasma and exosomes samples were determined using the 2^−ΔΔCt^ method relative to endogenous control (miR-1228) [37], and tissue samples to RNU6B (U6).

### Statistical analysis

Mann-Whitney test was used to compare the different expression levels of miRNAs between ESCC patients and NCs, and Wilcoxon test was used to compare the miRNA expression of paired tissue samples. One-way ANOVA or χ^2^ test was applied to evaluate clinical characteristics among different groups and their association with miRNAs. Multiple logistic regression analysis was used to establish the miRNA signature. ROC curves and the area under the ROC curve were used to evaluate the value of the identified miRNA or the miRNA signature in detecting ESCC.

SPSS16.0 software (SPSS Inc., Chicago, IL, USA) and GraphPad Prism 5 (GraphPad Software, USA) were used for statistical analyses and graphs. A two-sided *P* value < 0.05 was considered as statistical significance.

## SUPPLEMENTARY MATERIALS FIGURES AND TABLES


