# Supplementary material for: A six-microRNA signature in plasma was identified as a potential biomarker in diagnosis of esophageal squamous cell carcinoma
Source: Oncotarget. 2017 Mar 23;8(21):34468–80. doi: 10.18632/oncotarget.16519 (PMC5470983; doi:10.18632/oncotarget.16519)
Supplement: Supplementary file 1 [file oncotarget-08-34468-s001.pdf]

A six-microRNA signature in plasma was identified as a potential biomarker in diagnosis of esophageal squamous cell carcinoma

Supplementary Materials

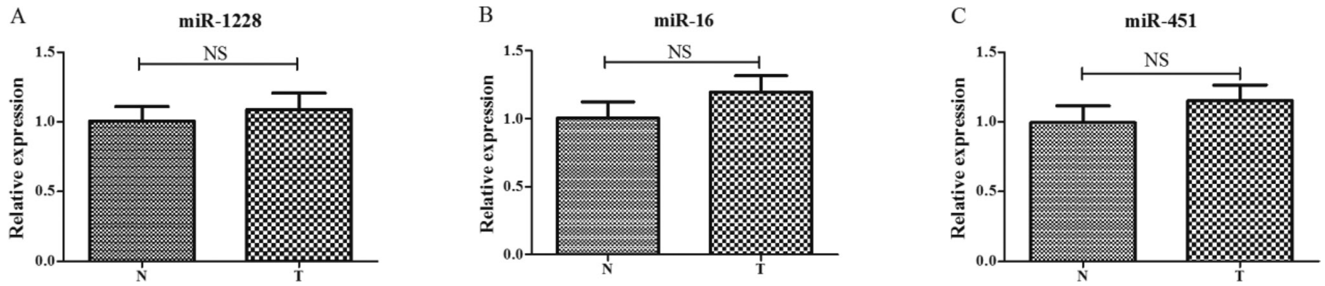

Supplementary Figure 1: Expression of miR-1228, miR-16 and miR-451 in plasma of ESCC patients and NCs (miR-1228: relative to sample volume; miR-16 and miR-451: relative to miR-1228). T: tumor; N: control; NS: not significant.

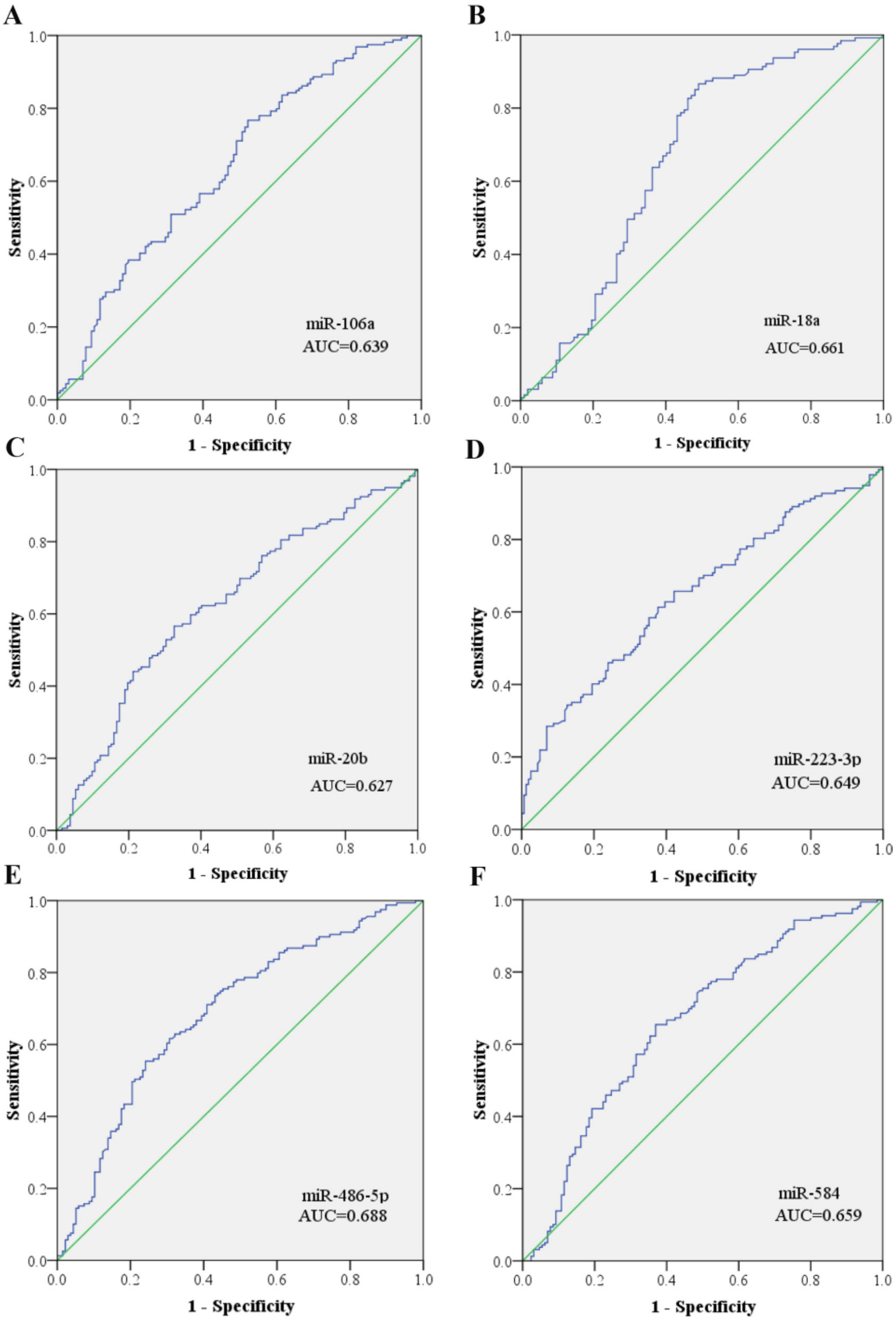

Supplementary Figure 2: Receiver-operating characteristic (ROC) curves of the six miRNAs to discriminate ESCC patients from NCs in the combined two cohorts of the training and the testing stage.

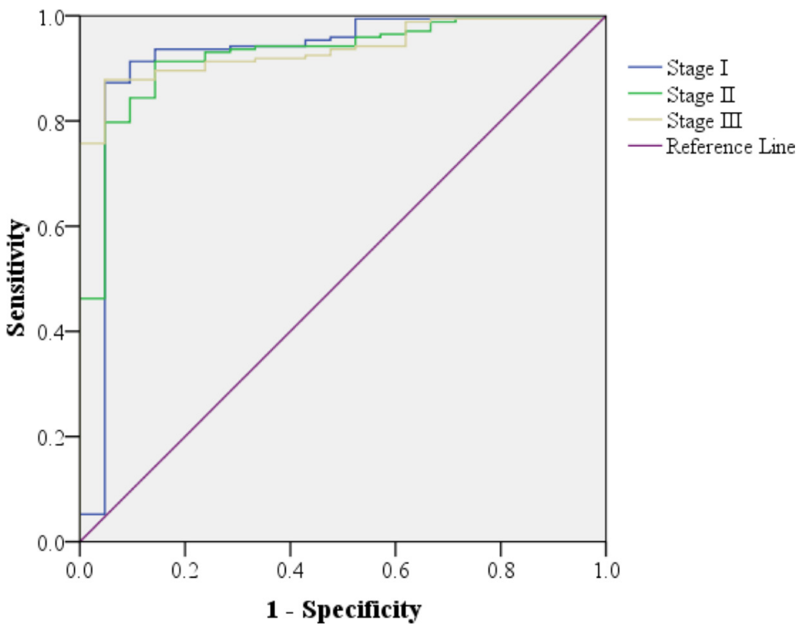

Supplementary Figure 3: Receiver-operating characteristic (ROC) curves of the six-miRNA signature to discriminate ESCC patients with stage I, II and III from NCs.

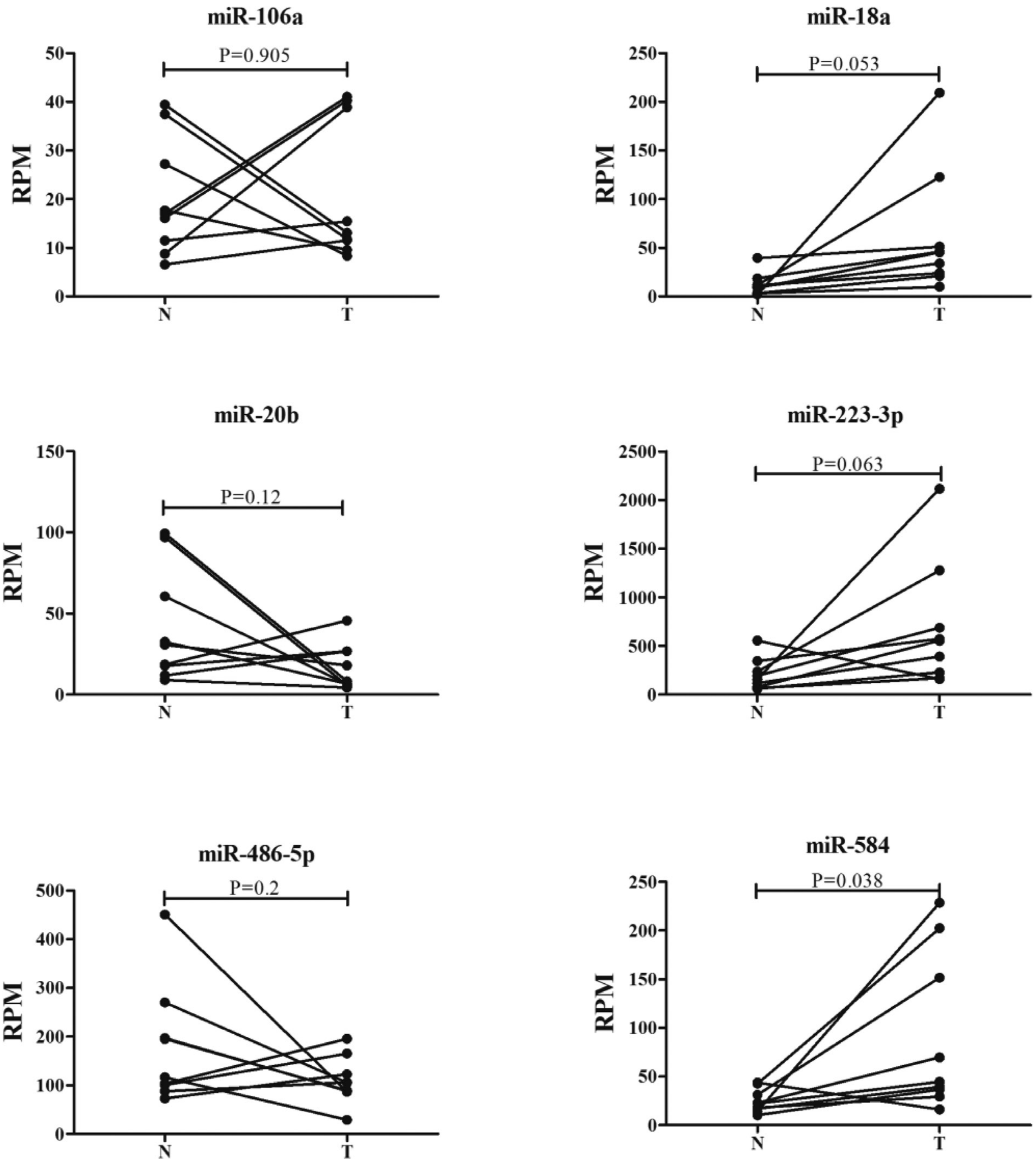

Supplementary Figure 4: Expression of the six miRNAs in the 9 pairs of ESCC and matched normal oesophageal tissues from TCGA database. T: tumor. N: control.

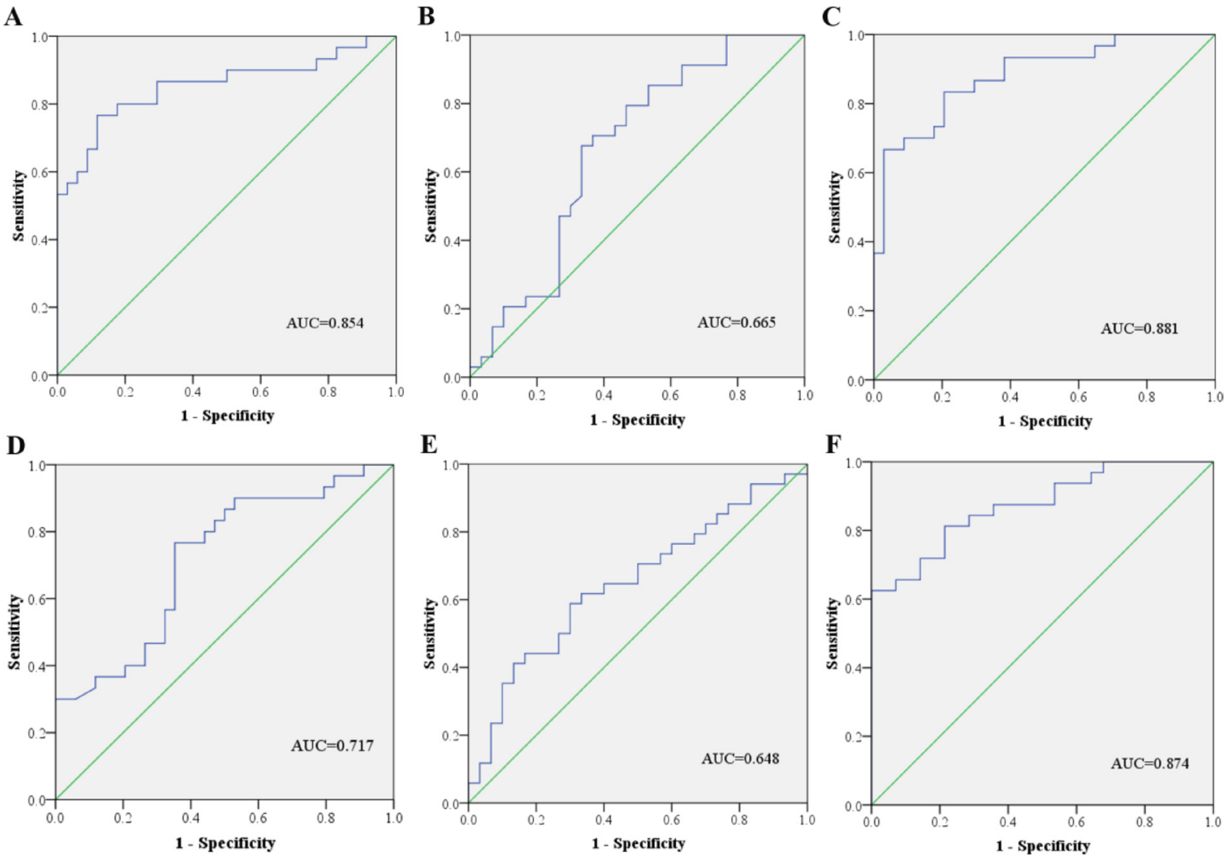

Supplementary Figure 5: The diagnostic capacity of exosomal miR-223-3p (**A**), miR-584 (**B**) and the signature of the two exosomal miRNAs (**C**) was evaluated and compared with plasma miR-223-3p (**D**), miR-584 (**E**) and the signature of the two plasma miRNAs (**F**) from the same subjects with receiver-operating characteristic (ROC) curves.

Supplementary Table 1: Differently expressed miRNAs in the screening phase

miRNA	Fold change		
	Pool 1	Pool 2	Mean fold
miR-25	1.73	2.02	1.87
miR-185	1.61	1.88	1.75
miR-205	1.57	1.83	1.70
miR-210	1.75	1.85	1.80
miR-215	2.81	3.28	3.05
miR-660	2.64	3.08	2.86
miR-106a	1.69	1.78	1.74
miR-140-3p	1.64	1.92	1.78
miR-18a	1.57	1.66	1.61
miR-19a	1.52	1.78	1.65
miR-20b	1.71	1.81	1.76
miR-324-3p	1.52	1.77	1.65
miR-486-5p	2.07	2.41	2.24
miR-500a	2.64	3.07	2.85
miR-502-3p	1.64	1.92	1.78
miR-532-3p	1.84	1.94	1.89
miR-92b	2.01	2.13	2.07
miR-154	-2.25	-6.20	−4.23
miR-584	-1.59	-3.29	−2.44
miR-125a-5p	-2.02	-3.02	−2.52
miR-127-3p	-4.76	-4.95	−4.85
miR-200c	-2.82	-9.97	−6.40
miR-223-3p	-2.14	-2.26	−2.20

Supplementary Table 2: Expression levels of the identified miRNAs from screening phase but not passed through the training stage and the testing stage (presented as mean ± SD; ΔCT, relative to miR-1228)

miRNA	Training stage				Testing stage			
	Cases	Controls	FC	*P* value	Cases	Controls	FC	*P* value
miR-185	6.86 ± 2.16	7.54 ± 2.36	1.61	0.019	7.32 ± 1.83	7.86 ± 2.94	1.45	0.13
miR-21	0.45 ± 1.22	1.09 ± 1.28	1.55	0.028	0.78 ± 1.23	1.22 ± 1.11	1.35	0.14
miR-125a-5p	7.5 ± 1.81	7.79 ± 2.37	1.22	0.37				
miR-127-3p	9.41 ± 1.45	9.48 ± 1.4	1.05	0.66				
miR-140-3p	6.44 ± 2.54	6.26 ± 2.53	0.88	0.96				
miR-154	5.14 ± 1.13	5.56 ± 1.39	1.34	0.14				
miR-19a	5.83 ± 4.48	6.2 ± 2.99	1.28	0.5				
miR-200c	10.19 ± 0.97	10.3 ± 1.41	1.07	0.27				
miR-205	9.01 ± 0.92	9.26 ± 1.33	1.19	0.54				
miR-210	6.2 ± 1.89	6.72 ± 2.32	1.43	0.17				
miR-215	4.65 ± 1.25	4.32 ± 1.46	0.79	0.19				
miR-25	2.42 ± 2.68	2.36 ± 2.52	0.95	0.69				
miR-324-3p	8.89 ± 4.24	9.49 ± 2.63	1.5	0.39				
miR-500a	6.66 ± 1.37	7.01 ± 1.56	1.28	0.37				
miR-502-3p	9.18 ± 4.22	9.84 ± 1.67	1.57	0.11				
miR-532-3p	9.44 ± 1.88	9.54 ± 2.35	1.07	0.51				
miR-660	5.86 ± 2.05	6.01 ± 2.37	1.11	0.6				
miR-92b	7.87 ± 1.83	8.49 ± 2.17	1.52	0.33				

FC: fold change.

Supplementary Table 3: Expression levels of the six miRNAs in the external cohort (presented as mean ± SD; ΔCT, relative to miR-1228)

miRNA	Cases	Controls	FC	*P* value
miR-106a	5.69 ± 2.41	6.94 ± 1.18	2.38	0.005
miR-18a	9.76 ± 2.55	11.12 ± 1.39	2.55	0.005
miR-20b	7.53 ± 2.52	8.53 ± 2.26	2	0.018
miR-223-3p	3.96 ± 3.41	2.25 ± 1.8	0.31	0.028
miR-486-5p	0.98 ± 1.93	1.59 ± 1.39	1.53	0.023
miR-584	8.89 ± 1.76	10.03 ± 1.21	2.21	0.002
